# Influence of the Tissue Collection Procedure on the Adipogenic Differentiation of Human Stem Cells: Ischemic versus Well-Vascularized Adipose Tissue

**DOI:** 10.3390/biomedicines12050997

**Published:** 2024-05-01

**Authors:** Pallabi Pal, Abelardo Medina, Sheetal Chowdhury, Courtney A. Cates, Ratna Bollavarapu, Jon M. Person, Benjamin McIntyre, Joshua S. Speed, Amol V. Janorkar

**Affiliations:** 1 Department of Biomedical Materials Science, School of Dentistry, University of Mississippi Medical Center, 2500 N State Street, Jackson, MS 39216, USA; 2 Division of Plastic Surgery, Department of Surgery, University of Mississippi Medical Center, 2500 N State Street, Jackson, MS 39216, USA; 3 Cancer Institute, University of Mississippi Medical Center, 2500 N State Street, Jackson, MS 39216, USA; 4 Department of Physiology and Biophysics, University of Mississippi Medical Center, 2500 N State Street, Jackson, MS 39216, USA

**Keywords:** flow cytometry, CD36, 3D spheroid, triglyceride, adipose tissue

## Abstract

Clinical and basic science applications using adipose-derived stem cells (ADSCs) are gaining popularity. The current adipose tissue harvesting procedures introduce nonphysiological conditions, which may affect the overall performance of the isolated ADSCs. In this study, we elucidate the differences between ADSCs isolated from adipose tissues harvested within the first 5 min of the initial surgical incision (well-vascularized, nonpremedicated condition) versus those isolated from adipose tissues subjected to medications and deprived of blood supply during elective free flap procedures (ischemic condition). ADSCs isolated from well-vascularized and ischemic tissues positively immunostained for several standard stem cell markers. Interestingly, the percent change in the CD36 expression for ADSCs isolated from ischemic versus well-vascularized tissue was significantly lower in males than females (*p* < 0.05). Upon differentiation and maturation to adipocytes, spheroids formed using ADSCs isolated from ischemic adipose tissue had lower triglyceride content compared to those formed using ADSCs isolated from the well-vascularized tissue (*p* < 0.05). These results indicate that ADSCs isolated from ischemic tissue either fail to uptake fatty acids or fail to efficiently convert those fatty acids into triglycerides. Therefore, more robust ADSCs suitable to establish in vitro adipose tissue models can be obtained by harvesting well-vascularized and nonpremedicated adipose tissues.

## 1. Introduction

The occurrence of obesity has steadily increased in recent decades and contributed to several diseases like type-2 diabetes, hypertension, and coronary heart disease in both men and women of all ages, races, and ethnicities [1]. By 2030, it is projected that approximately 60% of adults around the world will be overweight or obese [2]. In individuals with obesity, the adipose tissue is the largest endocrine organ because it can account for almost half the body weight [3]. Thus, in such individuals, minor metabolic changes in adipose tissue can impact the function of the entire body. Better knowledge of the adipose tissue structure and function is therefore critical to develop novel strategies that would work at the cellular level and reduce the deleterious effects of obesity. Mesenchymal stem cells are commonly used for adipose-related in vitro studies. Within this cell category, adipose-derived stem cells (ADSCs) are becoming popular as a result of their easy isolation from the adipose stromal vascular fraction (SVF), ease of adipose tissue accessibility, stem cell quality, and pluripotency. ADSCs also possess in vitro proliferative potential along with multilineage differentiation capacity [4,5,6].

The use of ADSCs in different in vivo and clinical settings is in rapid expansion. For instance, the application of ADSCs in in vivo animal models stimulates neovascularization in soft tissue transfer procedures [7,8]. ADSCs may have an important role in chimeric or prefabricated flaps for complex or three-dimensional surgical reconstructions in trauma patients or after cancer excision. In addition, ADSCs are thought to reduce ischemia/reperfusion injury, which may positively impact the management of burn injuries, acute kidney injuries, myocardial infarction, chronic nonhealing wounds (e.g., diabetic ulcers), and pressure ulcers and ischemic limbs [9,10,11,12,13].

Even though the clinical applications of ADSCs appear to be beneficial and safe, there is no current consensus on optimization of specimen collection and ADSC isolation or the appropriate dose, administration route, and treatment duration [14]. Rodbell and colleagues pioneered the technique to isolate ADSCs from rat epididymal fat [15]. The initial isolation procedure has undergone various modifications for isolation from human tissue. Current methods of cell isolation rely on collagenase digestion of the tissue to loosen the cells from the extracellular matrix and selecting the plastic-adherent ADSCs [16]. There is no definitive data on whether to use freshly isolated autologous SVF cells or in vitro expanded ADSCs because freshly isolated ADSCs and cultured ADSCs do not share the same phenotype and functions [17,18,19,20,21,22]. In addition to the physiological factors, numerous nonphysiological conditions are experienced during the current fat tissue harvesting procedures of abdominoplasty and liposuction that may affect the recruitment, proliferative potential, gene expression, and final reprogramming capability of the ADSCs. These factors include the considerable time of ischemia and hypoxia along with the effect of local medications (i.e., epinephrine, lidocaine, sodium bicarbonate, etc.). For instance, lidocaine has deleterious effects on ADSCs in a dose- and time-dependent manner regarding cell viability, proliferative capacity, and gene expression [23,24,25].

In this study, we aim to assess the differences between the stem cell populations isolated from adipose tissues harvested using the current method (which yields ischemic and chemically manipulated tissue samples) and a novel well-vascularized, nonchemically treated tissue harvest procedure. We hypothesized that an ADSC population obtained from “nonpremedicated and well-vascularized” adipose tissue has the potential to create more robust cell subsets in terms of lineage commitment (e.g., adipogenesis) and generate better in vitro culture models. This novel approach will determine how crucial procedural steps in clinical operational settings may negatively impact the current practice of ADSCs harvesting. To show the differences in marker-profile and differentiation-potential between ADSCs isolated from well-vascularized and ischemic samples, we have focused on the adipogenic differentiation. Current anti-obesity drugs mainly act like an appetite suppressor, have limited efficacy, and often come with significant side effects. To address this gap, researchers are turning to in vitro adipose culture models [26,27,28,29]. The evaluation of the effect of ADSCs origin would offer insights into adipocyte behavior under fat-laden conditions, crucial for developing more effective obesity treatments and combating its cardiovascular consequences.

## 2. Materials and Methods

### 2.1. Sample Collection

**Figure 1:**
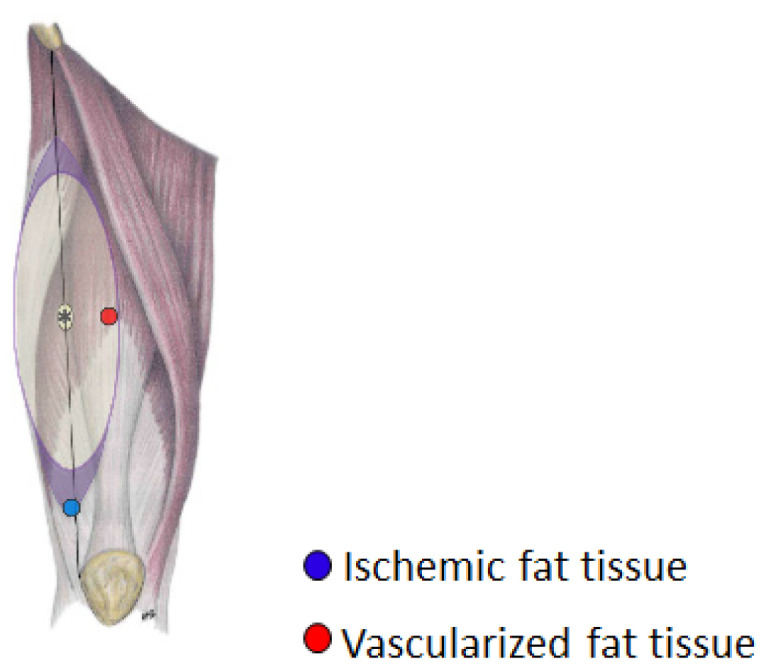
Site of adipose tissue collection during anterolateral thigh (ALT) free flap surgery. Well-vascularized tissue was harvested within the first 5 min of the initial surgical incision while ischemic tissue was harvested from the more distal part of the flaps.

Adipose tissue samples were harvested from adult male and female patients (n = 4 per group) undergoing anterolateral thigh (ALT) free flap surgeries (Figure 1). The donor characteristics are summarized in Table 1. All donors had no other comorbidities. The following samples were collected as approved by the University of Mississippi Medical Center Institutional Review Board (Approval # 2012-0004, Initial approval: 16 March 2012, Most recent annual approval: 24 June 2023):

1. Well-vascularized tissue: Adipose tissues were obtained at the beginning of the surgery (within the first 5 min of the initial surgical incision). These tissue samples were in a well-vascularized, nonpremedicated condition.
2. Ischemic tissue: Adipose tissues were obtained after the ALT flaps were fully harvested from the more distal part of the flaps where the blood supply was considered deprived.

### 2.2. ADSC Isolation and Maintenance

For the vascularized tissues, samples were collected and placed in the media immediately and transported to the laboratory for isolation. The ischemic tissue samples were obtained after the surgeries were complete (typically, 3 h after the vascularized sample collection). The ADSC isolation process lasts between 3 and 4 h. Consequently, the ischemic tissues experienced a total of 6–7 h of ischemic conditions. ADSCs isolated from the adipose tissues were cultured and differentiated following Turner et al. [6]. Briefly, the tissue was minced into small sections, digested in collagenase I (1 g L^−1^ in PBS) for 45 min at 37 °C and filtered through a 100 µM cell strainer. Pre-adipocyte media (1:1 DMEM:Ham’s F12 with 10% calf serum) was added to the filtrate and centrifuged at 1200× *g* for 5 min. The cell pellet was suspended in an erythrocyte lysis buffer for 10 min before centrifugation at 1200× *g* for 5 min. The pellet was resuspended in pre-adipocyte media and filtered through a 70 µM cell strainer. The filtrate containing SVF cells including ADSCs were cultured on tissue culture polystyrene dishes in pre-adipocyte media at 37 °C and 5% CO_2_, with media changes every 2–3 days. Cells were used for experiments between their third and sixth passage.

### 2.3. Cell Morphology

The morphology of ADSCs isolated from well-vascularized and ischemic tissues were examined using an Olympus IX 81 optical microscope. Images were captured at three different locations per culture plate using a Hamamatsu digital camera connected to Slidebook image acquisition software (Slidebook 4.2.0.10, Olympus, Center Valley, PA, USA).

### 2.4. Flow Cytometry

Flow cytometry measurements were conducted using mouse antihuman fluorochrome-conjugated monoclonal antibodies of CD29, CD31, CD36, CD44, CD59, CD90, CD105, CD106, CD117, and CD271. All antibodies except CD90 were purchased from BD Pharmingen™, while CD90 was purchased from eBioscience™, Invitrogen. Briefly, 100,000 ADSCs were washed twice with 1× mouse serum, resuspended in 1× mouse serum, and fluorochrome-conjugated antibodies were added per manufacturers’ protocols. After 30 min incubation, the cell–antibody conjugates were washed twice with 1× mouse serum and analyzed with a multicolor Beckman Coulter Gallios, B5-R1 configuration flow cytometer and Kaluza v2.1 software. Control gates were set with matched labeled isotype control IgG antibodies.

### 2.5. Coating Tissue Culture Plates

Elastin-like polypeptide (ELP) was produced in-house using genetically engineered *Escherichia coli* and conjugated with polyethyleneimine (PEI) following Turner et al. [30]. Then, 24-well tissue culture polystyrene plates were coated by adding 200 µL of 5 mol% ELP-PEI solution in deionized water (5 g L^−1^) per well and dried at 37 °C for 48 h.

### 2.6. ADSC Spheroid Formation, Differentiation, and Maturation

Fifty thousand ADSCs per well were seeded on the ELP-PEI coated 24-well plate and incubated at 37 °C, 5% CO_2_ for a 72 h period undisturbed to allow spheroid formation. ADSCs were then supplemented with differentiation media (1:1 DMEM:Ham’s F12 media with 1 µM dexamethasone, 0.5 mM IBMX, 0.1 U mL^−1^ insulin, 1 µM indomethacin, and 100 U penicillin/100 µg streptomycin mL^−1^) for 72 h [6]. After differentiation, cells were exposed to maturation media (1:1 DMEM:Ham’s F12 media with 10% fetal bovine serum, 0.2 U mL^−1^ insulin, 100 U penicillin/100 µg streptomycin mL^−1^) for up to 10 days. The cells were formed into spheroids and then differentiated into adipogenic lineage for two main reasons. First, this method avoids an extra trypsinization step required if the cells were to be differentiated first as a monolayer and then plated to form spheroids. Second, spheroid formation facilitates cell–cell interactions and cell–matrix communication. This architecture mimics the three-dimensional microenvironment found in vivo. Our prior work has shown that the spheroid culture can promote superior cell differentiation [6,30].

### 2.7. Measurement of Spheroid Size

Spheroids were imaged using an Olympus IX 81 optical microscope with a Hamamatsu digital camera and Slidebook image acquisition software (Slidebook 4.2.0.10, Olympus, Center Valley, PA, USA). ImageJ digital analysis software (https://imagej.net/ij, accessed on 22 January 2024) was used to measure the spheroid sizes on a minimum of 50 spheroids per condition. 

### 2.8. Biochemical Measurements

ADSC spheroids were collected after 3 days in differentiation media (i.e., day 0 of maturation media, M0) and 10 days in maturation media (M10) by aspiration and centrifuged for 2 min at 2000 rpm. Spheroids were then suspended in PBS and lysed using a Branson Digital Sonifier 450 (Danbury, CT, USA) for 1 min at 10% amplitude. All assays were performed in triplicates according to manufactures’ protocols. DNA and protein content were evaluated using the CyQuant DNA assay and BCA total protein assay, respectively (ThermoFisher Scientific, Waltham, MA, USA). Intracellular triglyceride (TG) content was measured using triglyceride and glycerol kit (Sigma-Aldrich, St. Louis, MO, USA).

### 2.9. Statistical Analysis

One-way ANOVA with the Games–Howell post hoc test for unequal variances was used for statistical data analysis. Data are shown as mean ± 95% confidence interval. Values with *p* ≤ 0.05 were deemed statistically significant.

## 3. Results

### 3.1. ADSC Morphology

**Figure 2:**
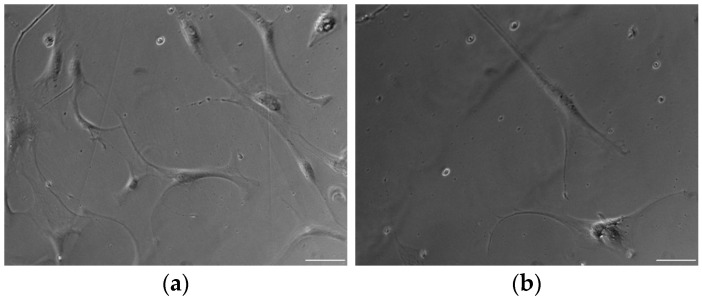
Optical microscopy images of ADSCs isolated from (**a**) well-vascularized and (**b**) ischemic adipose tissues. Scale bar = 100 µm.

Overall, there were no observable differences in the morphology of the ADSCs isolated from the well-vascularized and ischemic tissues (Figure 2). The cells of both types were spindle-shaped and elongated with a fibroblastic morphology, a characteristic feature of stem cells.

### 3.2. Flow Cytometry

**Figure 3:**
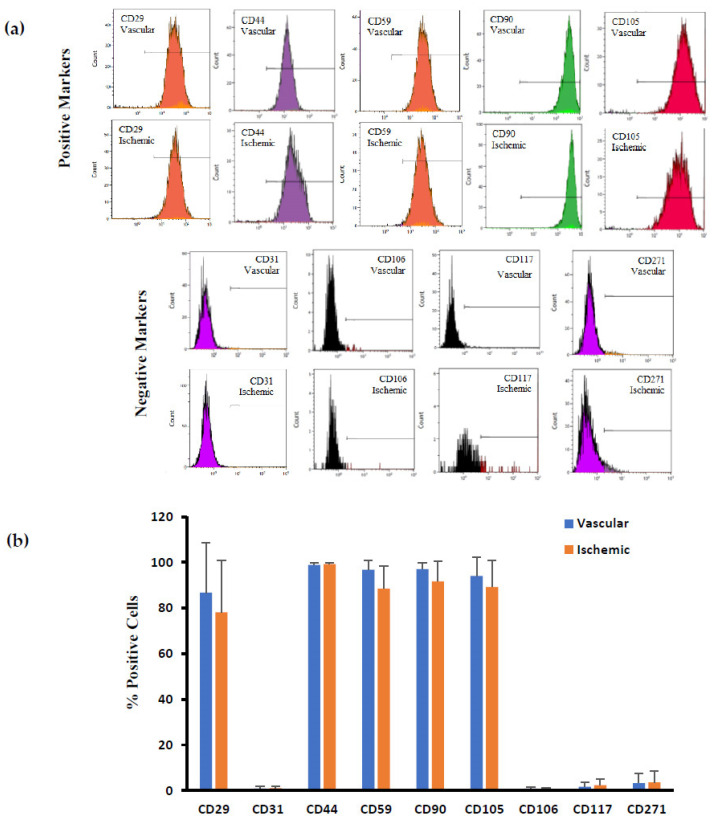
(**a**) Representative flow cytometry histograms of ADSCs isolated from well-vascularized and ischemic adipose tissues. (**b**) Quantitative measurement of the various CD markers. There were no statistically significant differences (*p* > 0.05) in the CD markers between the ADSCs isolated from the well-vascularized and ischemic tissues.

ADSCs isolated from the well-vascularized and ischemic adipose tissues positively immunostained with the conventional mesenchymal stem cell surface markers of CD29, CD44, CD59, CD90, and CD105 (Figure 3). ADSCs isolated from well-vascularized tissues exhibited the following percentage of positive cells for the indicated surface antigens (Figure 3b): CD29 (β 1 integrin), 86.7 ± 21.9%; CD44 (hyaluronate receptor), 98.9 ± 1.0%; CD59 (Protectin), 96.7 ± 4.0; CD90 (Thy1), 97.0 ± 2.8%; and CD105 (endoglin), 94.1 ± 8.0%. The corresponding values for ADSCs isolated from ischemic tissues were CD29, 78.1 ± 22.6%; CD44, 99.3 ± 0.4%; CD59, 88.7 ± 9.8; CD90, 91.7 ± 8.9%; and CD105, 89.3 ± 11.5%. Both types of ADSCs stained negative with CD31 (PECAM1) and CD106 (VCAM1) markers. A high degree of homogeneity was observed for both positive and negative surface markers on the ADSCs isolated from multiple donors (*p* ≤ 0.05). A negligible expression was noted for CD117 (NGFR) and CD271 (LNGFR) for the ADSCs isolated from well-vascularized tissues (2.1 ± 2.1% and 3.4 ± 4.2%, respectively) and those isolated from ischemic tissues (1.6 ± 1.3% and 4.0 ± 4.8%, respectively). Overall, there were no statistically significant differences (*p* > 0.05) in the abovementioned CD markers between the ADSCs isolated from the well-vascularized and ischemic tissues.

**Figure 4:**
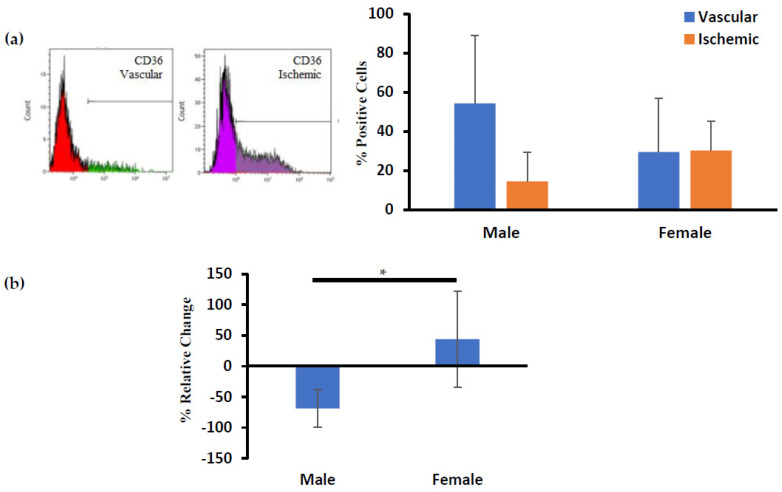
(**a**) Quantitative measurement of CD36 marker for ADSCs isolated from well−vascularized and ischemic adipose tissues of male and female patients. (**b**) The relative change in the CD36 marker for ADSCs isolated from well−vascularized and ischemic adipose tissues of male and female patients. Error bars indicate 95% confidence intervals. * *p* ≤ 0.05.

Interestingly, there was a relative change in the CD36 (FAT) marker, which is an integral surface protein involved in fatty acid uptake, for ADSCs isolated from well-vascularized and ischemic tissues of male and female patients (Figure 4). ADSCs isolated from well-vascularized male tissues had 54.3 ± 34.6% CD36 positive cells, while the ADSCs isolated from the ischemic male tissues had 14.6 ± 14.9% CD36 positive cells. ADSCs isolated from well-vascularized female tissues had 29.5 ± 27.4% CD36 positive cells, while the ADSCs isolated from the ischemic female tissues had 30.3 ± 14.8% CD36 positive cells (Figure 4a). Furthermore, we calculated the percent change in the CD36 expression of the ADSC isolated from ischemic versus well-vascularized tissues using the following formula:

$$Percent\;change=\frac{{\left(CD36\;Expression\right)}_{Vascular\;ADSC}-{\left(CD36\;Expression\right)}_{Ischemic\;ADSC}}{{\left(CD36\;Expression\right)}_{Vascular\;ADSC}}\times 100$$

The percent change in the CD36 expression of the ADSC isolated from ischemic versus well-vascularized tissues for male patients was 68.9 ± 30.5% lower while there was no such change for female patients (*p* < 0.05) (Figure 4b).

**Figure 5:**
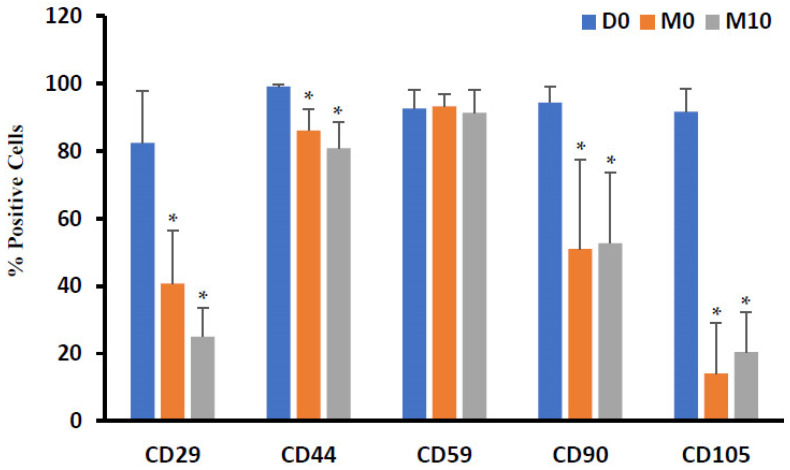
Quantitative measurement of various stem CD markers for undifferentiated ADSCs (D0), three-dimensional (3D) spheroid aggregates after 3 days of differentiation (M0) in adipogenic lineage, and ADSCs spheroid differentiated to adipocytes and matured for 10 days (M10). * *p* ≤ 0.05 compared to D0.

The stem cell markers for well-vascularized and ischemic tissues for men and women were systematically monitored following spheroid formation (D0 to D3), following 3 days of exposure to cell differentiation media (D3 to M0), and following 10 days of maturation into the adipogenic lineage (M0 to M10) (Figure 5). CD29 expression at D0, M0, and M10 were observed to be 82.4 ± 5.4, 40.8 ± 15.7, and 25.0 ± 8.5, respectively. CD44 expression at time points D0, M0, and M10 were observed to be 99.1 ± 0.5, 86.1 ± 6.5, and 80.8 ± 7.7, respectively. CD59 expression at time points D0, M0, and M10 were observed to be 92.7 ± 5.5, 93.3 ± 3.7, and 91.3 ± 6.8, respectively. CD90 expression at time points D0, M0, and M10 were observed to be 94.4 ± 4.7, 51.0 ± 26.4, and 52.7 ± 21.0, respectively. CD105 expression at time points D0, M0, and M10 were observed to be 91.7 ± 6.9, 14.1 ± 15.0, and 20.5 ± 11.9, respectively. The CD29, CD44, CD90, and CD105 expression on D0 was significantly different from M0 and M10 (*p* ≤ 0.05).

### 3.3. Size Distribution of Differentiated ADSC Spheroids

**Figure 6:**
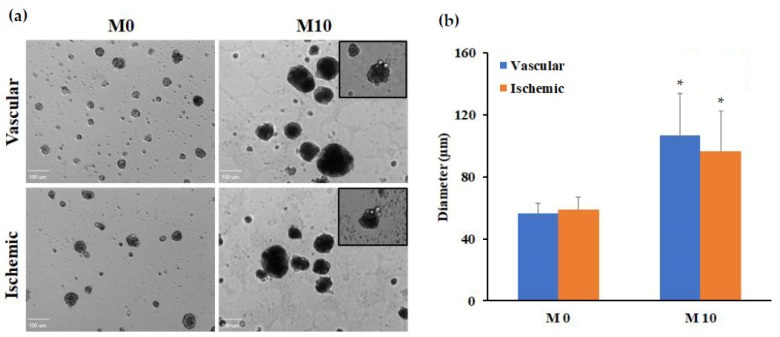
Spheroid formation of ADSCs isolated from the well-vascularized and ischemic tissues atop the ELP-PEI coated surface. (**a**) Bright field morphology, scale bars = 100 µm. The insets show unilocular fat deposits. (**b**) Quantitative measurement of the ADSC spheroid sizes (n > 50). Error bars indicate 95% confidence intervals. * *p* ≤ 0.05 between M0 and M10 timepoints for the same tissue source.

ADSCs cultured on ELP-PEI coated surfaces formed small three-dimensional (3D) spheroid aggregates during the first 3 days of culture. No significant variation in spheroid morphology or organization were seen between the spheroids prepared using the ADSCs isolated from the well-vascularized and ischemic tissues (Figure 6a).

With an increase in maturation time, the size of the initially formed small spheroids progressively increased, attaining a larger spheroid size by 10 days (M10) in both groups. The adipocyte spheroids after 10 days of maturation mostly had unilocular fat droplets (Figure 6a inset). Measurements taken from micrographs indicated that at the beginning of the maturation phase (M0), spheroids prepared using the ADSCs isolated from the well-vascularized tissues had an average diameter of 56.3 ± 6.7 µm and the corresponding values for the ADSCs isolated from the ischemic tissues were 58.8 ± 7.8 µm (*p* > 0.05). These spheroids gradually increased in size to 106.7 ± 27.1 µm and 96.6 ± 26.1 µm, respectively, over the 10-day maturation period (Figure 6b; *p* ≤ 0.05 for M10 versus M0 values; *p* > 0.05 between groups on the same day). Overall, the average spheroid diameter increased ∼2-fold over the 10-day maturation period, which is equivalent to a nearly 8-fold increase in the spheroid volume.

### 3.4. Biochemical Characterization

**Figure 7:**
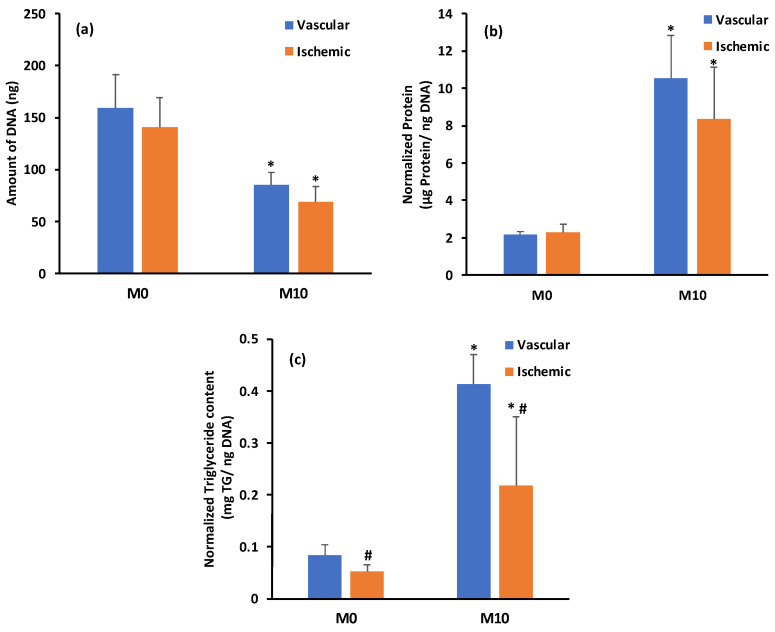
(**a**) DNA quantification; (**b**) normalized protein content; (**c**) normalized triglyceride content of spheroids prepared using the ADSCs isolated from the well-vascularized and ischemic tissues after 3 days of differentiation (Day 0 of maturation, M0) and 10 days of maturation (M10). Error bars represent 95% confidence intervals. * *p* ≤ 0.05 for M10 versus M0 values of the same group; # *p* ≤ 0.05 between groups on the same day.

The DNA content decreased significantly in both groups by the end of the 10-day maturation period (Figure 7a). The spheroids prepared using ADSCs isolated from well-vascularized and ischemic tissues had an average DNA content of 159.6 ± 31.8 ng and 140.5 ± 28.8 ng, respectively, on day M0 in maturation media (*p* > 0.05). These DNA content values gradually decreased over 10 days in maturation media to 85.2 ± 12.0 ng and 68.9 ± 14.8 ng, respectively, implying loss of spheroids due to media changes (*p* ≤ 0.05 for M10 versus M0 values; *p* > 0.05 between groups on the same day). Overall, there were no statistically significant differences (*p* > 0.05) in the average DNA content of the two groups at M0 and M10 days, indicating that the retention of spheroids over the 10-day maturation period was similar in both groups. 

Protein content normalized to DNA was stable in both groups over the 10-day maturation period (Figure 7b). On day M0, spheroids prepared using the ADSCs isolated from the well-vascularized and ischemic tissues had a normalized protein content of 2.2 ± 0.1 µg protein/ng DNA and 2.3 ± 0.4 µg protein/ng DNA, respectively (*p* > 0.05). After 10 days in the maturation media, the average protein content values were 10.5 ± 2.3 µg protein/ng DNA and 8.3 ± 2.8 µg protein/ng DNA, respectively (*p* ≤ 0.05 for M10 versus M0 values; *p* > 0.05 between groups on the same day). Increase in protein content over this period of time indicated the higher metabolic activity of the ADSCs in the spheroid configuration leading to adipogenic maturation.

Triglyceride (TG) content normalized to DNA is indicative of adipogenic differentiation and maturation to adipocytes. In comparison to day M0, both groups accumulated triglycerides over the 10-day maturation period (Figure 7c). The spheroids prepared using the ADSCs isolated from the well-vascularized and ischemic tissues had an average normalized triglyceride content of 0.08 ± 0.02 µg TG/ng DNA and 0.05 ± 0.01 µg TG/ng DNA, respectively, on day M0 (*p* ≤ 0.05). After 10 days in the maturation media, the average normalized triglyceride content values were 0.4 ± 0.1 and 0.2 ± 0.1 µg TG/ng DNA, respectively (*p* ≤ 0.05 for M10 versus M0 values; *p* ≤ 0.05 between groups on the same day). Overall, the average normalized triglyceride content increased ∼5–6-fold over the 10-day maturation period.

## 4. Discussion

Increasing evidence indicates that the changing environmental conditions cause changes in ADSCs response to proliferation, differentiation, migration, lipogenesis, and apoptotic susceptibility [31]. Here, we investigated if ADSCs from adipose tissue under ischemic conditions undergo dysfunctional alterations generating unique immunophenotypic profile and subsequent adipogenic differentiation compared with the ADSCs from a well-vascularized environment. Flow cytometry analysis of cell surface markers is an excellent technique to determine and differentiate between the type of cells isolated and to identify the expressed surface antigens that may have downstream functionality. This is the first study that examines differences in surface marker expression for ADSCs isolated under well-vascularized and ischemic conditions from same individuals. This identification and analysis are particularly relevant because these cells are a promising tool for cellular regenerative therapies, and the variation in surface antigen expression due to environmental or related factors may cause different protein expression on these cells, which may affect their usability in regenerative medicine. 

Benefits of 3D aggregates or spheroids over 2D monolayer cultures has been thoroughly investigated in recent times and results show increased expression of adipose-specific genes like PPAR-γ, CCAAT/enhancer-binding protein (C/EBP-α), and adiponectin along with enhanced triglyceride accumulation [32,33,34]. Moreover, 3D spheroids of adipocytes represent similar morphological character as seen in native adipose tissue. We have previously shown that cells seeded atop an ELP-PEI surface adopted spheroidal configuration and the cells better expressed lineage specific markers compared to 2D culture systems [4,6,30]. Our lab has shown that spheroid culture improves metabolic profile compared to typical monolayer cultures and spheroid growth correlates to better function [16,30]. In this study, we seeded the ADSCs on an ELP-PEI coated surface to induce them to form 3D spheroids and allow the cells to differentiate toward the adipocyte lineage. The cells isolated from well-vascularized and ischemic adipose tissues were analyzed per the guidelines set by the International Society for Cellular Therapy (ISCT) for stem cell identification and were confirmed to be ADSCs [35]. In our study, a similar positive expression profile for ADSC surface markers CD29, CD44, CD59, CD90, and CD105 was observed when compared to Zavan et al., Pachón-Peña et al., and Tucker et al. (Figure 3) [31,36,37]. A similar profile for the mesenchymal stem cells markers was also observed by De Francesco et al. for nonenzymatically extracted adipose derived stem cells [38]. While the isolated ADSCs on D0 expressed significant amount of CD29, CD44, CD59, CD90, and CD105, differentiation and maturation of the ADSCs along the adipogenic lineage resulted in significant reduction in the expression CD29, CD44, CD90, and CD105 (Figure 5). CD29 or integrin β1 is involved in the interaction of cells with the ECM proteins such as collagen, laminin, and fibronectin. CD44 is a type I transmembrane glycoprotein that binds hyaluronic acid (HA) in most cell types, which later plays a role in cell migration, cell–cell, and cell–matrix adhesion. CD90 or Thy-1 is a cell surface protein widely used as a stem cell marker that has speculated roles in cell–cell and cell–matrix interactions. As these CD stem cell markers play a major role in cell adhesion, the decrease in their expression can be contributed to either the formation of spheroids or differentiation of ADSCs to adipogenic lineage. CD59 is membrane bound glycoprotein that regulates complement mediated cell lysis. As reported by Festy et al., CD59 is homogeneously expressed by both precursor cell and mature adipocytes [39]. The similar expression of CD59 by both ADSCs on D0 and mature adipocytes on M10, thus agrees with Festy et al. CD105 (endoglin) is a type I membrane glycoprotein that functions as a receptor for TGF-beta ligands and is highly expressed in vascular endothelial cells. Interestingly, MSCs isolated from the adipose tissue have been shown to express CD105 at low levels when freshly isolated but become increasingly CD105+ upon culture passages. 

Our results for negative expression of CD31 also matches with Huang et al. and confirms absence of endothelial cells [40]. Calabrese et al. and Mifune et al. reported that the proliferation efficiency and trilineage differentiation of CD271+ stem cells isolated from adipose tissue is higher than those isolated from bone marrow [41,42]. Kohli et al. also illustrated that CD271+ MSCs significantly accelerated osteochondral wound healing with reduced vascularization when injected into athymic mice [43]. We found that the proportion of CD271+ cells were low, but similar in ischemic tissue compared to well-vascularized tissue (Figure 3). Recently, ISCT and International Federation for Adipose Therapeutics and Science (IFATS) have put forward additional markers for ADSC identification; positive expression profile for CD36 and negative for CD106 that clearly distinguishes them from bone marrow MSCs [44]. ADSCs isolated under the well-vascularized and ischemic conditions fit into this category because they were CD36 positive and CD106 negative (Figure 3 and Figure 4).

Currently, there are no FDA-approved anti-obesity drugs on the market that directly target the adipose tissue [26]. To identify an optimal drug candidate, an in vitro adipocyte model must closely mimic in vivo tissue structure. These models will provide insights into fat absorption, metabolism, and lipolysis mechanisms, aiding in the identification of promising drug candidates for further testing. These advancements not only decrease dependance on animal testing but also pave the way for innovative approaches to targeting adipocyte responses. Therefore, we differentiated the ADSCs isolated from well-vascularized and ischemic samples into adipocytes. The spheroids prepared using the ADSCs isolated from the well-vascularized and ischemic tissues showed a significant increase in size as well as lipid accumulation over the 10-day maturation period, suggesting their successful differentiation to adipocytes (Figure 6 and Figure 7). However, spheroids prepared using the ADSCs isolated from the ischemic tissues had a lower triglyceride content compared to the spheroids prepared using the ADSCs isolated from the well-vascularized tissues (Figure 7c). The behavior of the spheroids prepared using the ADSCs isolated from the ischemic tissues during 10 days of adipogenic maturation (lower triglyceride content) may be due to the ischemic/hypoxia condition experienced during the surgical harvest of the ADSCs, which may induce a more long-term metabolic dysfunction resulting in a less efficient commitment to follow adipogenic differentiation and, therefore, less effective fatty acids uptake and intracellular lipid metabolism. One such trigger may be the CD36-dependent autophagic pathway that maintains the cell homeostasis. It is well known that CD36 promotes the fatty acid uptake in adipocytes and other differentiated cells. However, little is currently known about the role of CD36 in multipotent undifferentiated ADSCs isolated from ischemic tissues. CD36 is a multiligand receptor contributing to glucose and lipid metabolism, immune response, inflammation, thrombosis, and fibrosis [45]. CD36 is also associated with macrophage foam cell development in atheromatous plaque formation, insulin resistance, oxidative stress (i.e., ischemia-reperfusion injury), and apoptosis/autophagy mechanisms [46]. Within CD36 functions, the CD36-dependent autophagic pathway is a cell survival mechanism that regulates cell homeostasis by modulating the mitochondrial dysfunction under ischemic conditions [47]. Consistent with these facts, it is not unreasonable to postulate that, in our ADSCs isolated from the ischemic tissues, the lower expression of CD36 plays a role in achieving the lower triglyceride content after 10 days of adipogenic maturation and the lower triglyceride content may be the result of increasing fatty acids catabolism to satisfy energy demands of the intracellular stress and therefore protect cells from apoptosis.

Limitations of our study include the relatively small number of samples used. We acknowledge that the limited sample size may impact the generalizability of our findings and the statistical power of our analyses. Additionally, assessments comprising of comparing cellular proliferation rates, determining ADSC yield from different types of adipose tissue, and evaluating multipotency through differentiation into osteocytes and cartilage cells will be valuable for a comprehensive understanding of ADSC characteristics. Even with these limitations, the implications of these findings for clinical practice are profound. This study will help develop a better understanding of the impact of tissue ischemia on ADSC functionality and could help in developing surgical strategies for adipose tissue harvesting. By optimization of tissue harvesting conditions to preserve tissue vascularity and minimize ischemic injury, clinicians may enhance the quality and functionality of isolated ADSCs for subsequent therapeutic applications. Furthermore, interpreting the molecular mechanisms underlying the observed functional differences may help develop interventions to improve ADSC function in ischemic conditions. This information could be used as a guide for development of novel strategies to enhance adipose tissue engineering and regenerative medicine approaches.

## 5. Conclusions

In this study, we compared variations in immunophenotypes and functionalities of ADSCs isolated from adipose tissues harvested within the first 5 min of the initial surgical incision, which is considered a well-vascularized, nonpremedicated condition versus tissues obtained after the ALT flaps were fully harvested and are from the more distal part of the flaps where the blood supply was considered deprived. The isolated ADSCs were confirmed to be mesenchymal stem cells. The ADSCs were cultured as 3D spheroids and after 10 days showed a significant increase in size as well as lipid accumulation, suggesting their successful differentiation to adipocytes. However, the spheroids formed using the ADSCs isolated from ischemic adipose tissue had a lower triglyceride content compared to those formed using the ADSCs isolated from the well-vascularized tissue. These results indicate that the ADSCs isolated from ischemic tissues either fail to uptake fatty acids or fail to efficiently convert those fatty acids into triglycerides. Elucidating the role of high CD36 expression in ischemic ADSCs may reinforce our hypothesis that the harvest of adipose tissues in well-vascularized and nonpremedicated conditions will yield the isolation of healthier ADSCs.

## Figures and Tables

**Table 1:** Characteristics of patients from whom the ADSCs were isolated. C = Caucasian, AA = African American or Black.

Patient #	Gender	Age	BMI	Race
1	Female	68	28.6	C
2	Female	74	30.9	C
3	Female	65	27.0	AA
4	Female	40	28.0	AA
5	Male	69	33.7	C
6	Male	58	33.3	C
7	Male	56	21.6	AA
8	Male	40	35.9	AA

## Data Availability

The data presented in this study are available on request from the corresponding author.
